# Regional variation and trends in prostaglandin analogue prescribing for glaucoma in England 2019–2024

**DOI:** 10.1038/s41433-026-04468-3

**Published:** 2026-04-22

**Authors:** David Grant Robinson, Angela Whitaker

**Affiliations:** 1 Cardiff University School of Optometry and Vision Sciences, Cardiff, UK; 2 Aneurin Bevan University Health Board, Caerleon, UK; 3 University Hospitals Bristol and Weston NHS Trust, Bristol, UK; 4 Moorfields Eye Hospital, London, UK

**Keywords:** Health care economics, Glaucoma, Drug therapy

## Abstract

**Background:**

Prescribing for glaucoma drops constitutes a large proportion of the NHS medicines budget for ophthalmology. NICE guidelines direct clinicians towards the prescription of a generic prostaglandin analogue (PGA) as a first-line topical hypotensive for lowering intraocular pressure. Between 2009 and 2018 the cost-effectivity of PGA prescribing at a national level in England was questionable. This study aimed to examine the variation and trends of PGA prescription at a regional level from 2019 to 2024.

**Methods:**

Using an online open-access source, monthly data relating to General Practitioner prescription of latanoprost, bimatoprost, travoprost and tafluprost were collected. Data gathered related to PGA prescription activity from each of the individual Clinical Commissioning Groups (CCGs) in England. Descriptive statistics and choropleth maps were produced to display outcomes.

**Results:**

Total PGA prescribing cost over 5 years was £166 M for 21.9 M prescriptions. A broad trend towards generic prescribing was identified. Branded and preservative free prescribing, in preference to a generic equivalent, resulted in an additional cost of approximately £54 M. Latanoprost was most readily prescribed in the South West and South East regions. Bimatoprost and travoprost were prescribed more in the North East and the East of England respectively.

**Conclusions:**

Regional variation in PGA prescribing exists in England. Further investigation is required to determine the primary cause for the reported differences. Reducing variations so that prescribing clinicians are directed towards the most cost-effective PGA choice has the potential for considerable cost savings to NHS budgets.

## Introduction

Globally, glaucoma is among the leading cause of irreversible blindness [1]. It is estimated that there are ~70 million people in the world with glaucoma and ~12.5 million are blind due to the disease [1, 2]. In the United Kingdom (UK), it is estimated that 4% of the population >50 years old have glaucoma, and a further 15% have ocular hypertension (OHT) or are a glaucoma suspect [3]. By 2035 the prevalence of glaucoma in the UK is expected to rise by 44% [4]. Despite advances in treatment, glaucoma continues to be the second leading cause of sight impairment in the UK, accounting for 10% of all sight impaired or severely sight impaired registrations [5].

The National Institute for Health and Care Excellence (NICE) introduced clinical guidelines in 2009 which aimed to standardise clinical practice concerning diagnosis and management of OHT and chronic open angle glaucoma (COAG) [6]. Since inception the guidelines have resulted in increased glaucoma case detection and referrals in England [7]. The 2022 revision recommends selective laser trabeculoplasty (SLT) as first line treatment for most patients with OHT or COAG. If a patient is unsuitable for or poorly responds to SLT, or in line with patient preference, offering a generic prostaglandin analogue (PGA) as first-line topical hypotensive is advised. In response to a pre-existing increase in use of costly preservative free (PF) glaucoma medication the previous 2017 revision recommended only offering PF prescriptions to patients with symptomatic ocular surface disease or known allergy to preservative.

Although the introduction of the guideline has resulted in a broad trend towards prescribing of generic PGAs, around half of glaucoma spending in England from 2009 to 2018 was accounted for by the use of branded and PF items [8]. In 2024/25 National Health Service (NHS) England spent over £11 billion on all medical prescriptions dispensed in the community [9]. This represented a 2% increase from 2023/24 and 6% of the annual NHS England outpatient prescription budget [10]. Within Ophthalmology, the reported cost to the NHS of prescribing ophthalmic prescriptions for glaucoma was £114 million in 2018 [8]. The mean cost of glaucoma treatment per patient has been reported to be between £400 and £500 per annum, up to a third of which relates to topical drug costs in glaucoma care [11, 12].

Between 2000 and 2012 a 67% increase in glaucoma prescriptions was reported in English primary care with half of all prescriptions issued being PGAs [13]. Although PGAs are a cost-effective option, cost-effective prescribing practice relating to PGA variants available from 2009 to 2018 in England has been questionable, particularly when considering the use of branded and/or PF items in place of a cheaper alternative [8].

Variations in prescribing have had significant financial consequences for NHS budgets. Across England, prior to the implementation of Integrated Care Systems, from 2013 until 2022 about 60% of the NHS budget was used by Clinical Commissioning Groups (CCGs) to commission most hospital and community NHS services [14]. It has been reported that over £30 million could be saved annually by prescribing generically (all glaucoma drug classes) over branded drugs, and that collectively, preservative free (PF) agents cost English primary care CCGs around £13 million in 2018 [8]. Generic latanoprost was significantly cheaper than other generic PGAs from 2009 to 2018 [8]. Evidence is lacking that any PGA is more effective than others at a level that is clinically significant for an individual patient [15]. Cost-effective prescribing practice therefore demands that prescribing clinicians factor in the relative costs of different PGAs when they are choosing the most appropriate PGA for their patients.

This study aimed to explore the geographical variation and trends of PGA prescription across the CCGs in England over a 5-year period from 2019 to 2024. This was done to assess cost-effectiveness of prescribing practice and alignment to UK clinical management guidelines at both a national and local level.

## Methods

Data was gathered from an online open-access source [16]. Openprescribing.net (Bennett Institute for Applied Data Science, Oxford, UK) collates the 700 million rows of anonymised general practitioner (GP) prescribing data that NHS England publishes monthly. A user can access up to 5 years of monthly data for any GP prescribed drug using its corresponding British National Formulary (BNF) code as an identifier. Drug codes were sourced from section 11.6 of the BNF treatment of glaucoma chapter. In alignment with current prescribing practice, data were extracted for the most commonly used PGAs in the UK (latanoprost, bimatoprost, travoprost and tafluprost). These PGAs were further subclassified as generic (preserved), branded (preserved) and PF (generic or branded) formulations. The following search criteria (BNF code) were used:

- Latanoprost: generic preserved (1106000L0AAAAAA), branded preserved (1106000L0BBAAAA) and PF (generic, 1106000L0AAACAC and branded, 1106000L0BCAAAB) 50 mg/ml eye drops.
- Bimatoprost: generic preserved (1106000AFAAABAB) and branded preserved (1106000AFAAAAAA) 100 mg/ml eye drops. Generic preserved (1106000AFAAAAAA), branded preserved (1106000AFBBAAAA) and PF (generic, 1106000AFAAACAC and branded 1106000AFBBACAC) 300 mg/ml eye drops.
- Travoprost: generic preserved (11060000AEAAAAAA), branded preserved (1106000AEBBAAAA) and PF (branded 1106000AEAAAB) 50 mg/ml eye drops.
- Tafluprost: generic preserved (1106000AKAAABAB), branded preserved (1106000AKBCAAAB/1106000AKBBABAB) and PF (branded 11060000AKAAAAAA) 15 mg/ml eye drops.

Data extracted included the number of topical PGAs prescribed monthly and over a 5-year period (December 2019 to November 2024) for each individual CCG (*n* = 106). Data for monthly cost of PGA prescriptions for each CCG were also collected. At the time of study, data for some branded preparations were not available; these data were collected during a post hoc analysis conducted in July 2025.

Data gathered were transferred and collated into a spreadsheet (Excel, Microsoft Corporation, Redmond, USA). Prior to analysis data relating to each drug were normalised relative to the total proportion of glaucoma drugs dispensed (dispensing proportion). This involved calculating monthly data of each drug per 1000 items for treatment of glaucoma within a given CCG. This was done by dividing the number of times a PGA was prescribed in a given month within the CCG by the total number of all glaucoma drugs prescribed in a given month within the same CCG, then multiplying by 1000.

Descriptive statistics were presented and choropleth maps were produced to enhance visualisation of CCG data. Formal analysis was performed using SPSS v.25 (IBM Corporation, New York, USA). Following normality testing (Shapiro-Wilk, *P* > 0.05) the Kruskal-Wallis test (*P* = 0.05) was used to analyse the significance of annual differences in the generic, branded and PF prescription rate of each drug in the 7 major NHS regions across England: East of England, London, Midlands, North East and Yorkshire, North West, South East and South West.

## Results

Data collection was completed over a 4-week period (February-March 2025). The raw dataset is provided in Suppl Table 1. For the 106 individual CCGs investigated there were no missing data relating to the 5-year study period. The total number of items issued over the study duration was 21.9 M, equating to a total cost of £166 M and an average cost of £7.57 per item. The total number of items (rounded to the nearest 10k) over the study period were: latanoprost (14.8 M), bimatoprost (5.3 M), travoprost (1.6 M) and tafluprost (240 K).

### Prescription rate

An overview of prescription trends is given in Table 1. When considering total number of items latanoprost was the only PGA that had a consistent increase per annum over the study period (increasing by 14%). The prescription rate for bimatoprost and travoprost decreased by 11% and 31% respectively. The tafluprost prescription rate remained static. Given the relatively small numbers of tafluprost items (1% of all PGA prescribing and 2% of PGA costs), no further analysis of tafluprost was undertaken.

**Table 1:** Total number of items (n) issued over the study period and associated cost (£) rounded to the nearest 10k (± standard deviation, SD).

	Latanoprost (±SD)		Bimatoprost (±SD)		Travoprost (±SD)		Tafluprost (±SD)	
	Items (*n*)	Cost (£)	Items (*n*)	Cost (£)	Items (*n*)	Cost (£)	Items (*n*)	Cost (£)
Year 1	**2.8 M** (±20k)	**20.8 M** (±300k)	**1.1 M** (±10k)	**18.7 M** (±160k)	**390k** (±10k)	**2.4 M** (±30k)	**50k** (±10k)	**710k** (±10k)
Year 2	**2.8 M** (±20k)	**16.3 M** (±170k)	**1.1 M** (±10k)	**13.8 M** (±200k)	**350k** (±10k)	**1.8 M** (±20k)	**50k** (±10k)	**680k** (±10k)
Year 3	**2.9 M** (±20k)	**15.5 M** (±150k)	**1.1 M** (±10k)	**11.6 M** (±100k)	**310k** (±10k)	**1.4 M** (±20k)	**50k** (±10k)	**690k** (±10k)
Year 4	**3.1 M** (±20k)	**17.7 M** (±200k)	**1.0 M** (±10k)	**11.5 M** (±100k)	**290k** (±10k)	**1.4 M** (±20k)	**50k** (±10k)	**710k** (±20k)
Year 5	**3.2 M** (±20k)	**18.4 M** (±130k)	**980k** (±10k)	**1.0 M** (±110k)	**270k** (±10k)	**1.3 M** (±20k)	**50k** (±10k)	**710k** (±20k)
Generic	**10.4 M** (±20k)	**38.9 M** (±280k)	**3.1 M** (±10k)	**28.7 M** (±240k)	**1.3 M** (±10k)	**4.6 M** (±30k)	**20k** (±10k)	**140k** (±10k)
Branded	**290k** (±10k)	**5.4 M** (±20k)	**870k** (±10k)	**14.5 M** (±80k)	**230k** (±10k)	**3.6 M** (±20k)	**10k** (±10k)	**90k** (±10k)
PF	**4.1 M** (±20k)	**44.4 M** (±190k)	**1.3 M** (±10k)	**22.4 M** (±60k)	**10k** (±10k)	**100k** (±10k)	**220k** (±10k)	**3.3 M** (±10k)
**Total**	**14.8 M** (±30k)	**88.7 M** (±480k)	**5.3 M** (±20k)	**65.6 M** (±370k)	**1.6 M** (±10k)	**8.3 M** (±50k)	**240k** (±10k)	**3.5 M** (±20k)

Data reporting generic and branded items relates to preserved formulations, preservative free (PF) data describes combined PF generic and branded variants.

### Cost Implications

A summary of cost implications is presented in Table 2. Over the study period generic preserved preparations, as opposed to branded preserved or PF equivalents, were prescribed most readily for all PGAs. With the exception of travoprost, PF preparations were more readily prescribed than branded preserved counterparts. Despite only contributing to 28% of all latanoprost prescriptions, £5.5 M more was spent on PF preparations of this drug than a preserved generic alternative. Similarly, a quarter of all bimatoprost prescriptions were attributed to PF formulations (34% of all spending on this drug).

**Table 2:** Overview of costs, rounded to the nearest 10k, across the 5-year study period for each prostaglandin analogue investigated.

	Drug	Cost (£)	Number of Items (n)	% of total PGA usage	Average cost per item (£)	% of total PGA prescribing cost
**Generic preserved**	Latanoprost	38.9 M	10.4 M	48	3.74	23
	Bimatoprost	28.7 M	3.1 M	14	9.26	17
	Travoprost	4.6 M	1.3 M	6	3.51	3
**Branded preserved**	Latanoprost	5.4 M	290k	1	18.62	3
	Bimatoprost	14.5 M	870k	4	16.67	9
	Travoprost	3.6 M	230k	1	15.65	2
**PF generic and branded**	Latanoprost	44.4 M	4.1 M	19	10.83	27
	Bimatoprost	22.4 M	1.3 M	6	17.23	14
	Travoprost	100k	10k	<1	10	>1

*PF* preservative free.

Over the study period the cost per unit of generic latanoprost and bimatoprost fluctuated greatly (Supplementary Fig. 1). At the start of the study generic latanoprost was still recovering from a cost spike in 2018. This was reported to be caused by temporary supply chain disruption that led to an abrupt, eightfold increase in the unit cost of the generic drug in the UK market [8]. Subsequently, the price steadily dropped and was at £4.18 per unit in November 2019. This continued to decrease to £1.71 per unit by December 2024. The generic bimatoprost tariff change was similar, peaking at £12.30 per unit in 2020 and dropping to £4.51 per unit by 2024. Generic travoprost cost changed less, with only a 25% decrease during the study period (£2.84 per unit in 2020 to £2.15 per unit in 2024). The mean price per unit of generic latanoprost, bimatoprost and travoprost over the study period was £2.46, £6.33 and £2.24. Overall, travoprost was consistently cheaper than the other generic PGA counterparts from 2019 until 2022 (£2.32 per unit on average). Latanoprost became the cheapest generic PGA from 2023 onwards (£1.88 per unit on average).

### Local prescription trends

**Fig. 1:**
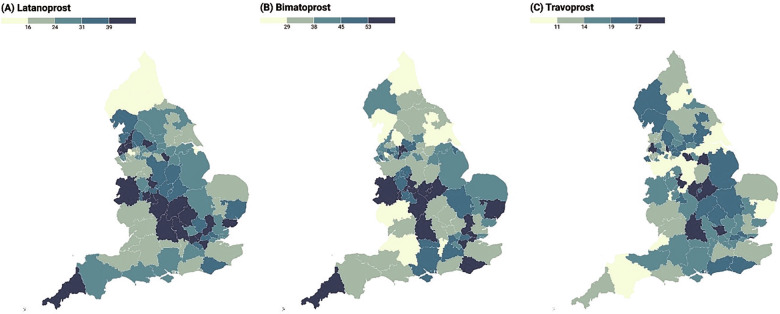
Prevalence of prescribing branded and preservative free prostaglandin analogue formulations. These choropleth maps show the regional variation in the proportion of branded or preservative free prescribing as a percentage of all PGA items within each CCG for **A** latanoprost **B** bimatoprost and **C** travoprost from Dec 2019 – Nov 2024. Values have been normalised per 1000 PGA items to account for population differences within each CCG. Increasingly darker colours represent a greater prevalence of branded preserved or PF prescribing as opposed to a generic preserved counterpart.

Figure 1 illustrates the prevalence of prescribing branded and PF formulations, as opposed to a generic counterpart, for each CCG. A third of all CCGs had a prescription rate of ≥30% for branded and PF latanoprost when prescriptions for these variants were added together. There was a strong prevalence for branded or PF bimatoprost prescribing with a quarter of all CCGs issuing generic versus branded or PF bimatoprost at a rate of 1:1. Branded or PF travoprost was less readily prescribed than other PGAs studied.

**Fig. 2:**
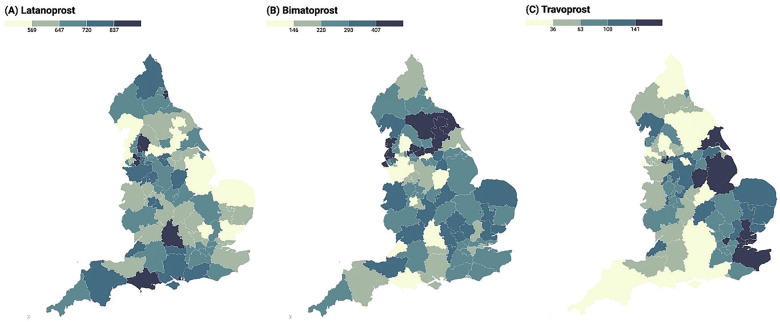
Prescription variation across individual CCGs. These choropleth maps show the regional variation of prevalence of prescribing latanoprost **A**, bimatoprost **B** and travoprost **C** from Dec 2019 – Nov 2024. Values have been normalised per 1000 PGA items to account for population differences within each CCG. Increasingly darker colours represent a greater prevalence of prescribing for each drug.

The prescription variation across individual CCGs is illustrated in Fig. 2. After adjustment for population, the CCG with the greatest mean number of overall generic, branded and PF latanoprost prescriptions ( ± SD) over the study period was Blackburn with Darwen (911 ± 128 per 1000). Information relating to the top 5 prescribing CCGs for each PGA is given in Suppl Table 2. The greatest number of bimatoprost and travoprost prescriptions ( ± SD) per 100 items were attributed to Wakefield (596 ± 94) and Manchester (191 ± 31) respectively. Conversely the CCGs with the least number of prescriptions ( ± SD) for each drug over 5 years were: Wakefield (latanoprost, 378 ± 28 per 1000), Sunderland (bimatoprost, 35 ± 6 per 1000) and North Tyneside (travoprost, 4 ± 2 per 1000).

The CCGs with the largest mean (normalised per 1000 PGA prescriptions) 5-year spend ( ± SD) on branded PGAs were Berkshire West (latanoprost, £140 ± 39), Heywood, Middleton and Rochdale (bimatoprost, £641 ± 115) and Southend (travoprost, £101 ± 16). The largest normalised 5-year spend on latanoprost and bimatoprost PF formulations were attributed to Greater Preston (£1130 ± 578) and Leicester City (£596 ± 146) respectively. Normalised PF travoprost spending was insignificant compared to other PGAs.

Latanoprost was most readily prescribed in the North West (Lancashire, Greater Manchester and Cheshire), North East (Tyne and Wear) and South (Hampshire and Dorset) regions. Hotspot locations outside of this included Oxfordshire, Barnsley, the Black Country, Brimingham and Solihull. PF latanoprost prescription was most prevalent in the North East (Newcastle Gateshead and Northumberland), Sussex and the South West (Devon, Cornwall and the Isles of Scilly) regions. Regions that prescribed the most latanoprost typically prescribed the least other PGAs e.g. the top three latanoprost prescribing regions (Blackburn and Darwen, Portsmouth and East Lancashire) were all positioned within the lowest quartile of prescribing locations for both bimatoprost and travoprost.

Bimatoprost was prescribed less readily than latanoprost in all CCGs except 3 (Wakefield, Blackpool and Southport, and Formby). The primary locations of bimatoprost prescription were identified as Yorkshire (Wakefield, Kirklees and York), Lancashire (Blackpool, Fylde and Wyre), Merseyside (Southport and Formby, Wirral and Sefton) and Greater Manchester (Heywood, Middleton and Rochdale). Regions that heavily prescribed bimatoprost were found to not readily prescribe latanoprost e.g. Wakefield was the lowest latanoprost prescribing region overall, similarly the second are third most active bimatoprost prescribing regions (Blackpool and Southport, and Formby) were also in the bottom 4 for latanoprost activity.

Travoprost was prescribed most readily in Essex (Thurrock, Castle Point and Rochford, Southend, Basildon and Brentwood), East Riding of Yorkshire (Hull) and Greater Manchester (Trafford). Travoprost prescription rate had little correlation with latanoprost and bimatoprost activity.

To further explore national variation, prescription trends within the 7 NHS regions (East of England, London, Midlands, North East and Yorkshire, North West, South East and South West) were assessed. Formal analysis (One-Way ANOVA) of the proportion of branded plus PF items issued showed significant differences (*P* < 0.050) in prescribing practice of latanoprost and bimatoprost. Notably the North East and South West regions prescribed less branded and PF preparations on average than the other regions. Branded and PF latanoprost was significantly prescribed more readily in London (*P* = 0.007) and the Midlands (*P* = 0.009) than in the North East region. Similarly, the East of England prescribed significantly more branded and PF bimatoprost than the North East (*P* = 0.023) and South West (*P* = 0.049).

## Discussion

This study has shown from that from 2019 to 2024 PGAs were prescribed readily in England at a cost to the NHS of £166 M. There were significant regional differences in the prescribing of PGAs for glaucoma and OHT. This corresponds to differences in the national expense of PGA prescribing which was not consistent across all regions in England.

During the 5-year study period there was a notable difference in average cost of the PGAs investigated, particularly between latanoprost and bimatoprost preparations. Generic preserved bimatoprost was consistently more expensive than all other generic PGA preparations studied. Preserved PGAs were consistently less expensive per item than PF (generic or branded) preparations of the same drug. NHS drug tariff prices during the study period also varied considerably, with generic latanoprost and bimatoprost preparations costing twice as much in 2019 as they cost in 2024. The average cost paid per item over the study period was greater than the mean 5-year cost of each drug. This is likely due to increased spending at higher costs earlier in the study period and inconsistency of drug cost at a local level.

Branded PGAs were considerably more expensive than generic counterparts e.g. branded latanoprost, bimatoprost and travoprost formulations were 5x, 2x and 4x more expensive than generic equivalents respectively. Branded prescribing, as opposed to generic, represented an additional cost of 13.5 M for all PGAs over the study period (latanoprost, 4.3 M; bimatoprost, 6.4 M; travoprost 2.8 M additional cost respectively). There may be legitimate reasons for prescribing a branded agent rather than generic, such as patient tolerance, administration issues or local availability. Regarding choice of drug, a meta-analysis by Li et al. did not find significant differences in effectiveness between different PGAs [15]. The study reported small differences in effect on IOP ( < 1 mmHg between bimatoprost, greatest effect, and travoprost, least effect) that were not clinically significant. Therefore a cost-effective prescribing rationale would not justify prescribing a first line preserved PGA that is more expensive than a different but cheaper preserved PGA. It would also not support switching from a cheaper first line PGA to a more expensive PGA to aim for improved IOP control unless there were supply or tolerance problems.

Preservative free PGA use was common throughout England. Spending on PF formulations, as opposed to generic preserved equivalents, resulted in an additional cost of ~£40 M over the study period (latanoprost, £29.1 M; bimatoprost £10.4 M; travoprost £65k additional cost respectively). The use of PGAs is guided by the NICE glaucoma diagnosis and management guidelines [6]. These guidelines utilised cost utility analysis to inform generic PGA use as a first line therapeutic treatment option. If more expensive PGAs such as branded or PF variants had also featured in the cost utility analysis it is possible that PGA recommendations, relating to class of medication or level of IOP to be treated, would be different. The guidelines currently recognise that the need for PF is a matter of clinical judgment, and that broadening of PF use may have legitimately increased over the past decade with changes in practice. This includes potentially more patients with ocular surface disease, use of PF to improve patient adherence to treatment, and more/earlier glaucoma surgery when surgeons may choose to reduce the ocular preservative load prior to surgery. However, as also previously reported, this study found significant regional variation in PF PGA prescribing practice [8]. The extent of the regional variation appears inconsistent with standardised use of an evidence based prescribing rationale.

National guidelines for glaucoma exist to help standardise and deliver equitable healthcare for glaucoma and OHT patients. Presently (and over the study period) most hospital formularies in England clearly specify that a generic PGA should be prescribed as a first line ocular hypotensive treatment. In alignment with this latanoprost was the most widely prescribed PGA investigated in this study. This aligns with other studies that have investigated glaucoma eye drop provision over the past decade in England [13, 17].

In agreement with the present outcome, the observational study by Heng et al also reported a regional variation in the overall glaucoma item prescriptions and a change in prescription rates from 2008 to 2012 in England [17]. Heng et al were able to correlate a modest proportion of these differences to age, ethnicity and male gender. However, as in this study, it was proposed that the variation was most likely because of local factors which were difficult to assess at a population level. They suggested influences such as individual risk and variations in eye care professionals’ prescribing practice were likely to have attributed to these disparities. Understanding fully why regional differences in prescribing exist is outside the remit of this study. Some of the factors which may dictate prescribing habits include variation in hospital formularies, differences in rates of glaucoma surgery or provision of laser treatment, differences in population requirements, influence of older research on prescribing habits, target IOP setting variation, industry influence and prescriber preference based on experience. Further research into this area would be beneficial to better understand what influences prescribing practice. Similarly, considering the increased use of SLT in glaucoma care an evaluation on the impact of ophthalmic laser provision, both as a primary and secondary treatment, on the usage of topical hypotensive drugs for glaucoma management would be valuable.

Presently, switching branded to generic medications (automatic generic substitution) is not mandatory in the UK. Although it is largely accepted that this controversial practice has the potential to significantly decrease drug costs, the financial benefit may be outweighed by the negative impact on quality of care [18, 19]. As switching medication has been linked with poorer adherence, a greater overall cost of care, associated with an increase in clinician visits or hospitalisations, has been reported [19]. In alignment with this it is prudent to recommend that clinicians managing glaucoma consider generic substitution with caution and on a case-by-case basis. The result of the present study suggests that significant savings can still be achieved through the prescription of generic PGAs from the outset as opposed to branded or PF alternatives.

The main strength of this study is the large, robust dataset which represents population data for the entirety of England over a sustained time period. Conversely, the data were geographically linked, as opposed to individually linked to patient records. This precluded calculation of actual patient numbers and the distinction of prescriptions dispensed for glaucoma from those dispensed for OHT. In addition, the study did not examine clinical reasoning behind PGA prescribing or reasons behind the regional variation in PGA prescribing. It is acknowledged that there will be individual patient factors responsible for a legitimate choice of a PF PGA preparation. It is therefore not possible to make assumptions about whether lower or higher rates of PF prescribing reflects alignment with evidence based prescribing practice.

The present study is also limited by its sampling method, restricted to GP prescribing data. A more comprehensive review of English prescribing practice would also consider hospital and private prescriptions, none of which are publicly available with the same level of detail. Given the frequency of hospital clinic and inpatient visits made by a typical individual with glaucoma, and the low proportion of English glaucoma care provided in the private sector, the present study is still likely to be representative of wider practice. It is also recognised that prescribing behaviour may be influenced by local factors such as drug availability, overall patient demographics, or service capacity. However, for branded versus generic PGAs, the study authors consider that clinical indications or local supply issues are unlikely to account for the large regional variation found in branded bimatoprost prescribing.

During the period studied, additional preparations of PGAs analysed became available. Post hoc analysis of the omitted branded formulations (*n* = 2 latanoprost, *n* = 4 bimatoprost) showed that spending on these preparations totalled ~£260k. Albeit important to acknowledge growing availability, their usage in small quantities has little impact on the overall result when compared to activity relating to the well-established counterparts used in the primary analysis.

## Conclusion

Glaucoma specialist clinicians who initiate PGA prescribing are recommended to follow NICE guidance by prescribing generically, to avoid switching between PGAs to improve IOP control, to take into account the range of costs between different PGAs, and to use PF only where clinically indicated. Data suggests that a minimum of 14 M could have been saved in England from 2019 to 2024 by preferential prescription of generic PGA preserved treatments in favour of branded preserved equivalents. Additionally, if generic latanoprost were adopted in preference to all PGA branded equivalents, the cost saving would have been in the region of 18 M. Preservative free PGA prescribing practice is also commonplace, with a 5-year cost implication (£40 M more than generic preserved equivalents) that seems out of alignment with the evidence base.

Although the majority of PGAs prescribed in England are generic, regional variation in PGA prescribing does exist, with some regions consistently opting for branded preparations in favour of generic equivalents. As there is no robust evidence base to support this preference, significant savings could be made if glaucoma clinicians considered the need for evidence based, cost-effective prescribing practice when initiating PGA based glaucoma care.

## Summary

### What was known before

- Glaucoma specialist clinicians in the United Kingdom who initiate prostaglandin analogue prescribing are recommended by NICE guidance to prescribe cost-effectively, using a generic drug preparation when clinically indicated.
- Between 2009 – 2018 although prostaglandin analogue prescribing in England was cost-effective compared with other therapeutic classes widely used to treat ocular hypertension and chronic open angle glaucoma, cost-effective prescribing practice relating to PGA variants available at a national level was questionable. Less was known about the variation and trends at a regional level.

### What the study adds

- From 2019 – 2024 in England branded and preservative free prescribing, in preference to a generic equivalent, resulted in an additional cost of approximately £54 M.
- Regional variation in prostaglandin analogue prescribing exists in England. Further investigation is required to determine the primary cause for the reported differences.
- Reducing variations so that prescribing clinicians are directed towards the most cost-effective prostaglandin analogue choice has the potential for considerable cost savings to National Health Service budgets.

## Supplementary information

Supplementary Figure 1

Supplementary Table 1

Supplementary Table 2

## Data Availability

Data available on reasonable request to corresponding author.
