# Supplementary material for: Regional variation and trends in prostaglandin analogue prescribing for glaucoma in England 2019–2024
Source: Eye (Lond). 2026 Apr 22;40(10):1483–9. doi: 10.1038/s41433-026-04468-3 (PMC13341770; doi:10.1038/s41433-026-04468-3)

**Suppl Figure 1**

Change in the annual number of items prescribed and mean cost per item for generic latanoprost (A), bimatoprost (B) and travoprost (C) over the study period.


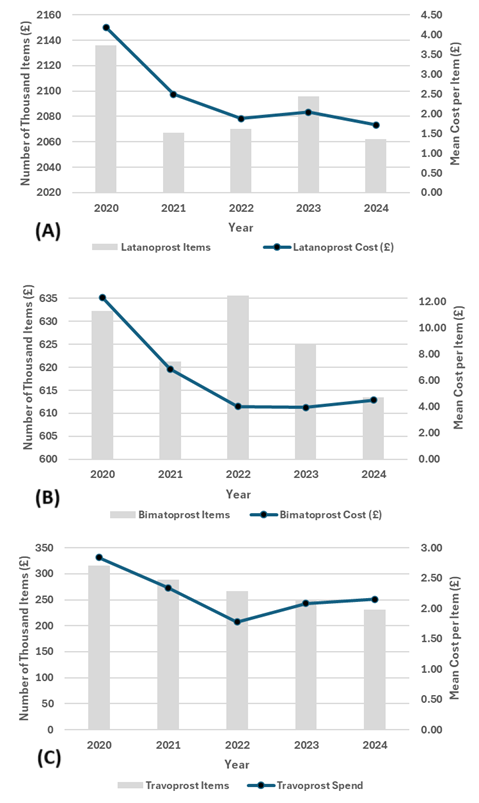

Supplement: Supplementary file 1 — Supplementary Figure 1 [file 41433_2026_4468_MOESM1_ESM.docx]
